# Long-Term Evaluation of Capsulotomy Shape and Posterior Capsule Opacification after Low-Energy Bimanual Femtosecond Laser-Assisted Cataract Surgery

**DOI:** 10.1155/2020/6431314

**Published:** 2020-09-23

**Authors:** Tommaso Verdina, Chiara Peppoloni, Lucrezia Barbieri, Maria Rosaria Carbotti, Bruno Battaglia, Rodolfo Mastropasqua, Gian Maria Cavallini

**Affiliations:** Institute of Ophthalmology, University of Modena and Reggio Emilia, Modena, Italy

## Abstract

**Purpose:**

To evaluate capsulotomy shape and posterior capsule opacification (PCO) during an 18-month follow-up for bimanual femtosecond laser-assisted cataract surgery (FLACS).

**Methods:**

74 eyes operated by a well-trained surgeon with bimanual FLACS technique using low-energy LDV Z8 (Ziemer Ophthalmic Systems AG, Port, Switzerland) were included in the study. The follow-up period was 18 ± 2 months. Another 91 eyes, which underwent standard bimanual microincision cataract surgery (B-MICS), served as a control group. In all cases, a BunnyLens AF (Hanita Lenses, Israel) intraocular lens was implanted in the bag. A digital image of the capsule with slit-lamp retroillumination was performed in all patients at 18 months of follow-up. Image analysis software (ImageJ) was used to evaluate the shape of the capsulotomy in terms of diameter, area, and circularity. PCO score was evaluated using EPCO 2000 software. Best corrected visual acuity (BCVA) and endothelial cell count (ECC) were evaluated before and after surgery at 1 and 18 ± 2 months.

**Results:**

At 18 months, mean capsulotomy diameter was 5.34 ± 0.21 mm while capsulorhexis was 5.87 ± 0.37 mm (*p* < 0.001) and the deviation area from baseline was 1.13 ± 1.76 mm^2^ in FLACS and 2.67 ± 1.69 mm^2^ in B-MICS (*p* < 0.001). Capsulotomy circularity was 0.94 ± 0.04 while capsulorhexis was 0.83 ± 0.07 (*p* < 0.001). EPCO score was 0.050 ± 0.081 in the FLACS group and 0.122 ± 0.239 in the B-MICS group (*p*=0.03). The mean BCVA improvement was significant in both groups, without a significant difference at 18 months. We noticed a statistically significant difference in endothelial cell loss at 18 months (FLACS 12.4% and B-MICS 18.1%; *p*=0.017).

**Conclusions:**

Bimanual FLACS is a safe and effective technique, as determined in a long-term follow-up. Capsulotomy shape presented higher stability and circularity in the FLACS group over the 18-month observation period. FLACS resulted in lower PCO scores and endothelial cell loss at 18 months in comparison to B-MICS standard technique.

## 1. Introduction

Age-related cataract is the second cause of moderate-to-severe vision impairment among the global population after uncorrected refractive errors. The strong impact of cataract in the public health justifies ophthalmological community interest in increasing precision and safety in cataract surgery [1].

In recent years, the use of a femtosecond laser has been introduced to assist the surgeon during cataract surgery. Even though femtosecond laser-assisted cataract surgery (FLACS) does not seem to show any significant difference with respect to refractive and visual outcomes when compared to standard phacoemulsification, recent studies showed better accuracy and reproducibility in the execution of corneal incisions, highly precise anterior capsulotomies, and nucleus fragmentation/liquefaction associated with a lower phacoemulsification energy and limited manipulation inside the eye [2–5].

The femtosecond laser LDV Z8 (Ziemer Ophthalmic Systems AG, Port, Switzerland) is a nonapplanating liquid patient interface femtolaser system characterized by high frequency and low energy [6]. It allows overlapping of very small laser spots creating a precise cut with minor gas bubbles and inflammation comparing to other femtolaser machines. It is entirely mobile, and its small dimensions allow surgeons to carry out the whole surgical operation in the same operating room, avoiding questionable patient transfer.

Bimanual microincision cataract surgery (B-MICS) is a variant of traditional coaxial phacoemulsification characterized by its incisional microinvasiveness (1.4 mm incisions) [7, 8]. The increased stability of the anterior chamber, the separation of the aspiration and the infusion probe together with the small instrument size, and greater visibility of the surgical field make it a safe and effective technique to be used in combination with a femtosecond laser [9–11].

It is well established that a perfect circular and properly sized capsulotomy is essential for stable results over time and better intraocular lens (IOL) position, especially for “premium” cataract surgery (multifocal and toric IOLs) [12]. Moreover, a better IOL overlapping with the anterior capsule has seen to cause less posterior capsule opacification (PCO) [13].

In a recent study, with a 3-month follow-up period, we demonstrated that the low-energy/high-frequency properties of the LDV Z8 laser pulse, combined with the overlapped pulse-pattern, resulted in highly continuous morphology of capsule edges. [14]. In addition, a higher apoptosis induction (Caspase-3 analysis) of the epithelial cells of the capsule edge has been demonstrated at confocal immunofluorescence in FLACS in comparison to manually performed capsulorhexis in standard cataract surgery [14, 15], which could reduce the incidence of PCO during the follow-up.

To our knowledge, currently there are no studies investigating PCO incidence and capsulotomy shape in FLACS with a low-energy/high-frequency femtosecond laser.

The aim of the study was to evaluate the shape of anterior capsulotomy and PCO incidence during an 18-month follow-up for bimanual FLACS with a low-energy femtosecond laser, comparing the results with a control group of standard B-MICS interventions. Secondary outcomes were best corrected visual acuity (BCVA) and endothelial cell count evaluated preoperatively and at 18-month follow-up.

## 2. Methods

We retrospectively reviewed the data of 165 eyes who underwent cataract surgery at the Institute of Ophthalmology of the University of Modena and Reggio Emilia between March 2016 and July 2018. Seventy-four eyes underwent bimanual cataract surgery using FLACS technique (FLACS); 91 eyes, operated with the B-MICS standard technique, were chosen as a control group. All surgeries were carried out by the same expert surgeon (GMC). Patients in both groups were similar in age, gender, and lens LOCS III score (Table 1). The study was approved by the Ethics Committee of the University of Modena and Reggio Emilia (Modena, Italy) and was conducted in compliance with the Declaration of Helsinki. Informed consent was obtained before surgery.

Exclusion criteria included eyes with previous surgery, complicated cataracts (e.g., hard cataracts, traumatic cataract, and pseudoexfoliation syndrome), insufficient mydriasis (<4 mm), concomitant eye pathologies (e.g., uveitis, glaucoma, corneal opacities, and diabetic retinopathy), low endothelial cell count (<1500 cells/mm^2^), and monocular patients.

A detailed clinical history evaluation was carried out on all the patients prior to surgery with best corrected visual acuity (BCVA) examination, anterior segment biomicroscopy, and fundus examination. Biometry was performed with IOL Master (Carl Zeiss Meditec, Jena, Germany) and corneal microscopy with a Noncon Robo-CA (Konan Medical Inc., Hyogo, Japan).

Patients applied nonsteroidal anti-inflammatory drug (NSAID) drops preoperatively 3 times a day for 3 days before surgery in both groups.

A consistent mydriasis was obtained before surgery with the instillation of atropine 1.0%, phenylephrine 10%, and cyclopentolate 1%, and a locoregional anaesthesia was carried out with peribulbar block (1.5 ml of lidocaine 2% and 1.5 ml of bupivacaine 0.5%). All interventions were performed using the same phacoemulsifying machine (Faros, Oertli Instruments AG, Berneck, Switzerland). Standard B-MICS and FLACS surgery using the bimanual technique with the LDV Z8 and IOL implantation through a 1.4 mm incision have been previously described [11].

FLACS capsulotomy diameter was 5.2 mm in all cases. The surgeon implanted the same model of hydrophilic acrylic BunnyLensAF IOL (Hanita Lenses, Israel) in all eyes.

BCVA and endothelial cell count (ECC) were evaluated after surgery at 1 and at 18 ± 2 months using the instruments previously described. In particular, ECC was evaluated by manually counting a group of cells and then providing a rapid morphometric automated endothelial analysis by the machine. The same experienced doctor carried out all these examinations.

Digital retroilluminated images of the pseudophakic anterior segments of the eyes were taken at day-1 and again at 18 ± 2 months postoperative follow-up visit using a camera connected to the slit lamp. Images of the capsule were taken following dilation of the pupil in all patients.

Image analysis software (ImageJ) was used to evaluate the shape of the capsulotomy.

ImageJ is biomedical imaging software for scientific image analysis, which is available as a free download from an open platform [16, 17].

**Figure 1:**
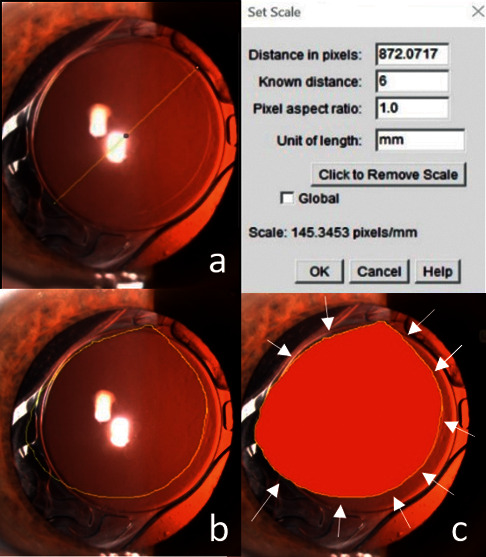
Anterior capsule opening analysis with ImageJ software. (a) Setting of the scale measuring the 6 mm IOL optic plate diameter as known true size. (b) Laying out of the contour of anterior capsule edge. (c) Software analysis (capsulotomy area indicated in red).

Capsulotomy/capsulorhexis analysis was carried out in three phases: firstly, the scale was deﬁned by measuring the size in pixels for a feature of known true size. In this case, the 6 mm IOL optic plate diameter was ﬁrst calculated on an image. The number of pixels that corresponded to 6 mm was calculated. Then, the contour of the anterior capsule edge was defined by the operator. Finally, through an automated analysis, the software calculated the major and minor diameter (mm), the area (mm^2^), and the coefficient of circularity (from 0 = lower circularity to 1 = perfect circularity). (Figure 1).

PCO score was evaluated using the computer-based software Evaluation of Posterior Capsule Opacification 2000 (EPCO2000) at 18 ± 2 months of follow-up. This software is based on the morphological assessment of PCO and allows a quantitative and qualitative evaluation of the amount of IOL surface affected by opacification, as previously described [18].

**Figure 2:**
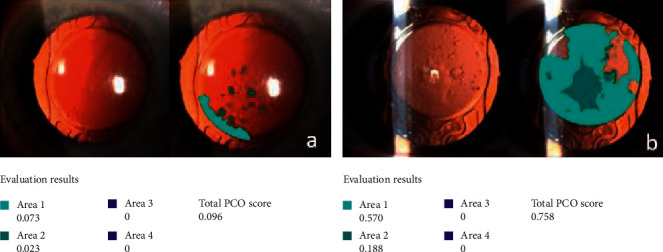
PCO analysis with EPCO2000 software in two study cases: (a) FLACS, patients (*n*), 41; (b) B-MICS, patients (*n*), 52. *Note.* Left pictures are from the slit lamp (native image), and right pictures are those from the correspondent software analysis (evaluated image). Evaluation results and legend for each single image are on the left.

The EPCO2000 software was used to evaluate each image providing a final PCO score for every eye examined. The PCO score for each eye is calculated by multiplying the density of the opacification, graded from 0 (none) to 4 (severe), by the fractional PCO area involved behind the entire IOL optics. Density areas were identified and marked interactively on the computer screen by the same expert observer who was blinded to the surgical procedure used (Figure 2).

The neodymium-yttrium-aluminum-garnet (Nd : YAG) laser capsulotomy rate was recorded.

An Excel database (Microsoft Excel 2010 and Microsoft Office Professional Plus 2010) was used to record all data; for data analysis, we used Stata 13.1 software (StataCorp LP, College Station, TX, USA) with Student's *t*-test and the Wilcoxon rank sum test. A post hoc power analysis was applied to verify the features of two groups. Statistical significance was indicated by a *p* value <0.05.

## 3. Results

Group A consisted of 74 eyes (40 right eyes and 34 left eyes) of 42 patients (24 males and 32 females); the average age was 74.83 ± 5.29 years.

Group B was made up of 91 eyes (52 right eyes and 39 left eyes) of 63 patients (22 males and 41 females); the average age was 75.94 ± 8.95 years.

The two groups were homogeneous and comparable for age and gender with no statistical and clinical differences (Table 1). No intraoperative complications were recorded. A BunnyLens AF IOL was implanted in all eyes. All IOLs were implanted in the capsular bag.

Demography of the study population is summarized in Table 1. Results in both groups are reported in Tables 2–4.

### 3.1. Continuous Curvilinear Capsulotomy/Capsulorhexis (CCC)

Concerning anterior capsule opening diameters, our analysis showed that FLACS capsulotomy presented lower changes in mean diameter and mean area over an 18-month follow-up than B-MICS capsulorhexis.

In particular, capsulotomy diameter variation was 0.14 ± 0.21 mm while that of capsulorhexis was 0.29 ± 0.19 mm (*p* < 0.001). We registered a lower mean area variation in FLACS (1.13 ± 1.76 mm^2^) than in B-MICS (2.67 ± 1.69 mm^2^) (*p* < 0.001).

**Figure 3:**
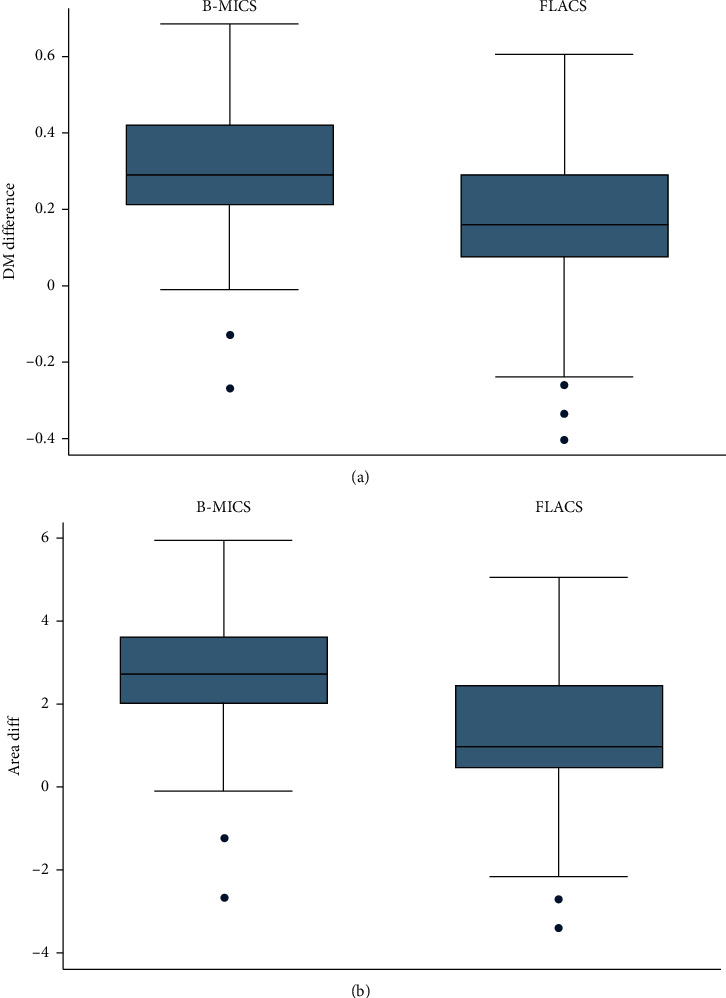
Box plot with whiskers indicating 95% confidence intervals for diameter (a) and area (b) variation in an 18-month follow-up period. DM = diameter.

Moreover, the circularity index at 18 months was 0.94 ± 0.04 in FLACS while 0.83 ± 0.07 in B-MICS (*p* < 0.001). All the results are shown in Table 2 and Figure 3.

### 3.2. PCO Incidence

PCO score in FLACS was lower (0.050 ± 0.081) than B-MICS (0.122 ± 0.239), and the difference was statistically significant (*p*=0.03). PCO was registered in 5 cases out of 71 (6.8%) for FLACS and 27 cases out of 91 (29.7%) for B-MICS.

**Figure 4:**
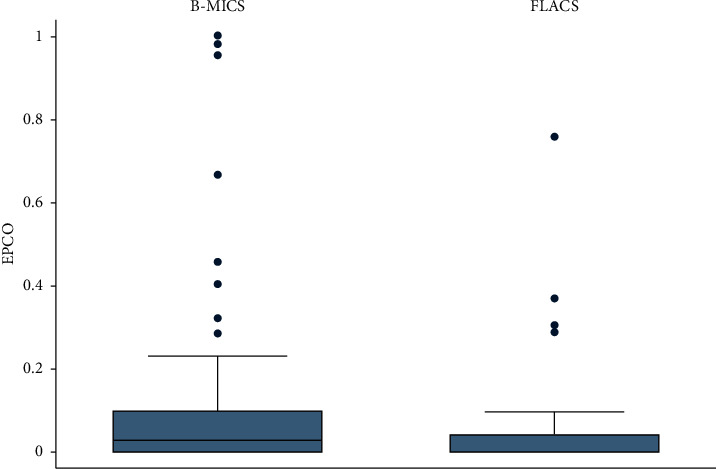
Box plot with whiskers indicating 95% confidence intervals for EPCO score variation in an 18-month follow-up period.

Nd : YAG laser capsulotomy at 18 months was necessary only in 1 case for FLACS and in 8 cases for B-MICS. Detailed results are reported in Table 3 and Figure 4.

### 3.3. Postoperative Results

In the FLACS group, at the 18-month follow-up mark, a mean BCVA improvement of 0.404 ± 0.346 LogMAR was observed and was statistically significant from baseline (*p* < 0.001). Similarly, in the B-MICS group, a mean BCVA improvement of 0.400 ± 0.261 LogMAR was observed (*p* < 0.001). However, the difference in the improvement of visual acuity between the two groups was not statistically significant (*p*=0.46).

Regarding the ECC, at the 18-month follow-up visit, a mean endothelial cell loss of 288 ± 424 cells/mm^2^ in Group A (12.4%) and of 443 ± 356 cells/mm^2^ in Group B (18.1%) was observed and showed a reduction that was statistically significant between the two groups (*p*=0.017) (Table 2).

## 4. Discussion

With the introduction of femtosecond laser technology, cataract surgery is experiencing a period of change and scientific fervor. The femtosecond laser does not only assist and facilitate cataract surgery but also standardizes some crucial steps: allowing precise and reproducible corneal microincisions, perfectly circular and centered anterior capsulotomies, and lens fragmentation, thus, ultimately leading to a reduction in ultrasound energy.

Recently, we published a paper about the safety and effectiveness of the combination of FLACS with bimanual technique [11]. However, the usefulness of FLACS is still debated since recent meta-analyses showed no statistically significant differences with standard manual cataract surgery in terms of visual and refractive outcomes and general complications [2–5]. Many surgeons maintain conflicting views on the effective large-scale spread of this surgical technique in the near future.

The interest of the scientific community is nowadays rather focused on the evaluation of long-term effects of FLACS as this procedure could have a positive influence on IOL stability and PCO incidence over time in comparison to the standard technique.

A recent study reported a more precise and stable capsulotomy in FLACS patients in a 12-month follow-up period using a high-energy/low-frequency femtosecond laser platform in comparison to a control group [19].

In this study, we investigated long-term (18 months) results obtained by an expert surgeon with bimanual FLACS technique with a low-energy/high-frequency femtolaser in terms of capsulotomy shape and PCO incidence. We also evaluated visual outcomes and endothelial cell loss at 18 months after surgery. We compared results with a control group. Up to date, there are no studies on a long-term evaluation of FLACS treated with a low-energy/high-frequency femtosecond laser.

Recent scientific investigations have proven that FLACS capsulotomies are more predictable, regular, and better-centered than manual ones, leading to safer surgery and a positive influence on visual recovery and patient satisfaction [20]. Moreover, the regularity of the capsulotomy/rhexis shape and size influences the position of the IOL and the predictability of the calculated power for the IOL [21, 22]. It is well known that if the CCC is too small, it can cause a hyperopic outcome caused by a posteriorly pushed IOL due to an excessive anterior capsular overlap. If it is too large, the IOL can be positioned too anteriorly resulting in a myopic shift. Moreover, if the capsulotomy is not well-centered, the IOL can be tilted causing astigmatism or a compromised retinal image [23]. In addition, a capsulotomy, which is centered on the optical axis of the lens with a diameter of 5.25 mm, optimizes prevention of PCO, consistency of effective lens position (ELP), and capsular strength [12].

In our study, we found that FLACS capsulotomy presented a significantly higher stability of the capsulotomy diameter and area at 18 months after surgery. Moreover, the FLACS group had a significantly higher circularity than B-MICS capsulorhexis.

Our findings are in line with results reported in the literature showing significant centration and stability of FLACS capsulotomy over time in comparison to standard phacoemulsification. In particular, Pathier et al. [19] evaluated the diameter of the rhexis, its centration, and the position of the IOL in 33 patients who underwent FLACS in one eye and traditional phacoemulsification in the other; they found that in the FLACS group, the laser capsulotomies were more precise, centered, and stable over time.

Similar results were found by Friedman et al., who showed higher symmetry, centering, and circularity in the FLACS group compared to the controls [24]. Berk et al. in a recent study evaluated 995 cases of FLACS and 883 cases of traditional phacoemulsification after three weeks from surgery, highlighting more precisely centered, circular, and reproducible capsulotomies in the first group [25].

High-energy/low-frequency femtolasers were used in the above mentioned studies. It is known that laser pulse energy influences the strength of the capsulotomies, which increases with decreasing the energy [24]. Previous research with the low-energy/high-frequency Ziemer Z8 showed, similar to our results, median circularity 0.98 [0.97–0.99] (*n* = 6) with FLACS, better accuracy when compared to manual capsulorhexis and importantly very smooth capsulotomy edges due to low energy [26]. In contrast to ours, the research was undertaken using human donor eyes and no follow-up examination was possible.

We also observed a better IOL overlapping with capsulotomy than in manual capsulorhexis. Complete overlap of the IOL optic by the anterior capsule edge is a well-known enhancement of the barrier effect to lens epithelial cell growth. Hollick et al. [27] have long ago reported and confirmed significantly less PCO with a capsulorhexis completely covering the edge of the IOL optic.

Posterior capsule opacification (PCO) remains one of the main long-term complications after cataract surgery, especially when hydrophilic IOLs are concerned, often seen as one of the most common causes of nonrefractive decrease in vision [28, 29]. In our study, PCO score and incidence were significantly reduced in the FLACS group compared to the standard phaco group.

In the literature, uncertain data about this controversial topic are found. Kovacs et al. observed a lower PCO incidence at 18–26 months in the femtolaser group in comparison to standard phacoemulsification [13]. On the other hand, some recent investigations reported results in contrast with this evidence [25, 30].

PCO reduction could be attributed to a well-centered capsulotomy or to the lens epithelial cell apoptosis at the margin of the capsulotomy as previously described [14]. With regards to cell apoptosis and loss, different reports exist, but all are in agreement that the low-energy minimizes cell loss and reduces peripheral damage along the capsulotomy [31–33]. Thus, the cell loss at the capsulotomy margin alone cannot explain the lower occurrence of the PCO in low-energy FLACS.

It is well demonstrated that FLACS procedures performed with high-energy lasers are associated with higher prostaglandin and cytokine concentrations and higher rates of anterior capsule damage [34], whereas reduced inflammation could also decrease the risk of PCO induction, and Liu et al. found only low amounts of interleukin (IL)-1*α* and −1*β* in the anterior chamber aqueous humour of patients who underwent FLACS with the low-energy Z8 [35]. The IL-1*α* of 0.5 ± 0.2 pg/ml was only slightly higher compared to manual surgery (0.05 ± 0.05 pg/ml), and the IL-1*β* showed almost similar values for the FLACS and manual surgery: 0.5 ± 0.3 pg/ml and 0.5 ± 0.4 pg/ml respectively. The numbers reported with high-energy lasers are up to 25.6 pg/ml [36].

Interleukin-1 receptor antagonist has been shown to suppress the proliferation of lens epithelial cells [37], and this might provide an explanation to our findings and support a favorable view of low-energy FLACS in the aspect of the occurrence of postoperative PCO [38]. For these reasons, we suppose that a low-energy femtosecond laser could give even more advantages in terms of a reduction of PCO occurrence in FLACS.

Regarding postoperative results, we registered a significant improvement in mean BCVA in both groups, as previously found for FLACS [2–5, 39], without any significant difference between the two groups. As for endothelial cell loss, the results obtained in our study showed a reduced loss in the FLACS group than in the control group, and this difference was statistically significant. This is likely due to the reduction in phaco energy, which has been shown to harm the endothelium. The positive impact of FLACS on the endothelium has been widely reported in the literature regardless of the femtolaser system used [11, 40–45] even though recently a few papers reported on no significant differences in endothelial cell loss between the two techniques [46, 47]. The present study reports on a longer follow-up period, and the results are obtained from a low-energy femtosecond laser, which is a first report according to our knowledge.

A limitation of our study was its retrospective nature: a randomized prospective clinical trial and another blind observer for PCO and capsulotomy evaluations should be preferred. Another limitation was that data collection at 18-month follow-up was not at that precise time-point for every patient but varied ±2 months. Moreover, endothelial cell loss was not assessed by a blind observer.

In conclusion, low-energy femtosecond laser technology in conjunction with the advantages of B-MICS technique shows good results in cataract surgery in a long-term follow-up. FLACS with LDV Z8 showed a more stable capsulotomy shape and a higher circularity, when compared to standard capsulorhexis, and registered a significantly decreased PCO score with a lower YAG laser incidence than in the standard phacoemulsification group. These findings have a significant impact on cataract surgery as stable capsulotomy influences the IOL centration. Furthermore, a low incidence of PCO reduces the need for a YAG-laser procedure, which is expensive and has related risks such as retinal tear or detachment. Moreover, a reduced endothelial cell loss in FLACS was also retrospectively confirmed in our 18-month follow-up study.

Further studies will be needed to confirm the data, in particular the analysis of visual outcomes after toric, multifocal, or trifocal IOL implantation or in complicated cases such as endothelial disease, weak zonules, or other ocular conditions in which overall reduced energy and mechanical manipulation should be needed Table 4.

## Figures and Tables

**Table 1:** Comparison of demographic data at baseline for both groups.

	Group A (FLACS)	Group B (B-MICS)	*p* value^*∗*^
Eyes *(n)*	74	91	—
Mean age *(y) ± SD*	73.19 ± 6.88	74.70 ± 8.32	*p* > 0.05
Right/left eyes *(n)*	40/34	52/39	*p* > 0.05
Male/female *(n)*	24/32 (56)	22/41 (63)	*p* > 0.05
Cataract grade (LOCS III)	2.37 ± 0.49	2.49 ± 0.50	*p* > 0.05

**Table 2:** CCC diameter and area, BCVA, and ECD results at baseline and 18 months for the two groups.

	FLACS			B-MICS		
	Baseline	18 months	Difference	Baseline	18 months	Difference
CCC diameter (mm)	5.20	5.34 ± 0.21	**0.14** **±** **0.21**	5.60 ± 0.40	5.87 ± 0.37	**0.29** **±** **0.19**
CCC area (mm^2^)	21.24	22.37 ± 1.69	**1.13** **±** **1.76**	24.93 ± 3.50	27.16 ± 3.75	**2.67** **±** **1.69**
BCVA (logMar)	0.420 ± 0.342	0.016 ± 0.065	**0.404** **±** **0.346**	0.443 ± 0.238	0.043 ± 0.020	**0.400** **±** **0.261**
Endothelial cell density (cells/mm^2^)	2290 ± 466	2007 ± 230	**288** **±** **424**	2448 ± 337	2005 ± 489	**443** **±** **356**

**Table 3:** PCO score and percentage of PCO cases and YAG laser in the two groups.

	FLACS (74)	B-MICS (91)
PCO score	0.050 ± 0.081	0.122 ± 0.239
Total cases with PCO	6.8% (5 cases)	29.7% (27 cases)
Nd : YAG laser	1.4% (1 cases)	8.8% (8 cases)

**Table 4:** Summary of study findings at 18 months with statistical significance of the comparison.

	FLACS	B-MICS	*p* value
CCC differential diameter (mm)	0.14 ± 0.21	0.29 ± 0.19	<0.001
CCC differential area (mm^2^)	1.13 ± 1.76	2.67 ± 1.69	<0.001
Circularity index (0-1)	0.94 ± 0.04\	0.83 ± 0.07	<0.001
Visual gain BCVA (logMar)	0.404 ± 0.346	0.400 ± 0.261	*p*=0.46
Endotelial cell loss (cell/mm^2^)	288 ± 424	443 ± 356	*p*=0.017
EPCO	0.050 ± 0.081	0.122 ± 0.239	*p*=0.03

## Data Availability

The data used to support the findings of this study are available from the corresponding author upon reasonable request.
